# Occurrence of multidrug-resistant methicillin-resistant *Staphylococcus aureus* among healthy farm animals: a public health concern

**DOI:** 10.1080/23144599.2019.1689630

**Published:** 2019-11-22

**Authors:** Khaled A. Abdel-Moein, Hala M. Zaher

**Affiliations:** Department of Zoonoses, Faculty of Veterinary Medicine, Cairo University, Cairo, Egypt

**Keywords:** MRSA, farm animals, multidrug resistance, public health

## Abstract

Methicillin-resistant *Staphylococcus aureus* (MRSA) is an emerging pathogen causing serious public health threats. This study was conducted to investigate the occurrence of multidrug-resistant MRSA among apparently healthy farm animals to shed the light on the potential role of these animals as a reservoir for such pathogen. For this purpose, 195 nasal swabs from apparently healthy farm animals (52 sheep, 51 goats, 47 cattle and 45 buffalo) were screened for multidrug-resistant MRSA. MRSA was isolated using a selective chromogenic medium and identified by colonial characters, Gram’s stain films, conventional biochemical tests, coagulase test, resistance to cefoxitin and amplification of *nuc* and *mec*A genes. The antimicrobial susceptibility testing profile was performed by disk diffusion method to identify multidrug-resistant MRSA. Of 195 samples, 7 yielded MRSA with an overall prevalence 3.6%, whereas the prevalence rates were 3.8%, 3.9%, 4.3% and 2.2% for sheep, goats, cattle and buffalo, respectively. All MRSA isolates were multidrug-resistant strains. The phylogenetic analysis of 2 *mec*A gene sequences from the obtained isolates revealed that both sequences were clustered in the same clade with those derived from human clinical cases from different countries to highlight the public health burden of such strains. The distribution of multidrug-resistant MRSA among all examined farm animal species being apparently healthy points out that farm animals could represent a potential reservoir for multidrug-resistant MRSA with public health implications.

## 1. Introduction

*Staphylococcus aureus* is a superbug pathogen causing serious diseases in humans and animals [1]. The first antibiotic used for curing severe *S. aureus* cases was penicillin but resistance to such drug soon appeared following its clinical use [2]. Researchers were seeking for new antibiotics to combat penicillin-resistant *S. aureus* strains; this led to development of methicillin [3]. Unfortunately, methicillin-resistant *S. aureus* (MRSA) was emerged in the UK in 1960 to tackle its use [4], since that time, MRSA became a major crisis in the human medicine worldwide. Moreover, MRSA goes beyond human being to invade animals whilst in Belgium, 1972, bovine mastitis was the first reported case of MRSA infection in animals [5]. Afterwards, several studies recorded MRSA infections among farm and pet animals making MRSA as an emerging veterinary pathogen [6–8], this gives an opportunity for increasing evidence of its zoonotic potential [9]. Because *S. aureus* is remarkable to acquire antibiotic-resistant determinants exhibiting resistance to multiple classes of antimicrobial agents [10,11], multidrug-resistant MRSA has become one of the most important current threats to the public health. After being well established in the health-care setting, MRSA found its way towards the community and now community-acquired MRSA has a mounting public health burden [11]. Given the serious threat of zoonotic MRSA transmission, the subsequent detection of multidrug-resistant MRSA among animals was of major concern [12,13]. Therefore, the current study was conducted in order to investigate the occurrence of multidrug-resistant MRSA among apparently healthy farm animals to shed more light on the potential role of these animals as a reservoir for multidrug-resistant MRSA.

## 2. Material and methods

### 2.1. Collection of samples

Nasal swabs from apparently healthy 195 farm animals (52 sheep, 51 goats, 47 cattle and 45 buffalo) were collected from different private farms in Giza governorate, Egypt. Each swab was placed in Cary-Blair transport medium and transferred in an icebox immediately to the laboratory for bacteriological examination.

### 2.2. Isolation and identification of MRSA

Samples were enriched overnight in 5 ml tryptone soy broth (TSB) containing 6.5% NaCl before plating on CHROMagar MRSA medium (CHROMagar, France) [14] and incubated at 37°C for 48 h. Pink to mauve colonies were subcultured on mannitol salt agar to obtain a pure culture. The colonies suspected to be *S. aureus* were identified by Gram’s stain, standard biochemical tests, haemolytic activity on blood agar and coagulase test according to Quinn et al. [15].

### 2.3. Molecular confirmation of S. aureus

Genomic DNA was extracted from isolates using the rapid DNA extraction protocol as described by Reischl et al. [16]. Molecular confirmation of *S. aureus* was carried out by detection of *S. aureus*-specific *nuc* gene using primers *nuc*1 and *nuc*2 and the amplification was done according to McClure et al. [17].

### 2.4. Phenotyping identification of MRSA and antimicrobial susceptibility testing

Antimicrobial susceptibility testing was performed using the disk diffusion method recommended by Clinical & Laboratory Standards Institute [18]. Eighteen antimicrobial agents were tested for antimicrobial susceptibility in all MRSA isolates. Penicillin G, oxacillin, cefoxitin, ceftaroline, gentamicin, chloramphenicol, clindamycin, azithromycin, erythromycin, ciprofloxacin, norfloxacin, tetracycline, doxycycline, linezolid, trimethoprim/sulfamethoxazole, rifampin, quinupristin/dalfopristin and nitrofurantoin were tested using Mueller–Hinton agar and commercially available disks (Oxoid, UK). The results of the antimicrobial susceptibilities of the analysed strains were scored according to the guidelines of CLSI 2018. MRSA isolates were phenotypically identified after resistance to cefoxitin whereas multidrug resistance was defined as a resistance to at least one agent in three or more antimicrobial categories [19].

### 2.5. Molecular confirmation of MRSA

**Figure 1.**
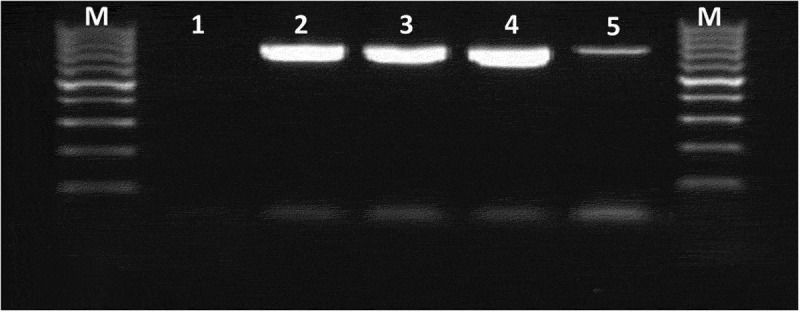
Molecular detection of *mec*A gene among multidrug-resistant MRSA strains. Lane M, DNA ladder 100 bp; lane 1 negative control; Lanes 2, 3, 4, 5 positive samples showed specific bands at 776 bp.

The molecular confirmation of MRSA was carried out through amplification of *mec*A gene. The PCR assay was carried out using forward (5ʹTGGCTCAGGTACTGCTATCCAC 3ʹ) and reverse (5ʹ AGTTCTGCAGTACCGGATTTGC 3ʹ) primers [20]. The amplification conditions used were initial denaturation at 94°C for 5 min, followed by 30 cycles of denaturation at 94°C for 30 sec, annealing at 60°C for 30 sec and extension at 72°C for 30 sec with a final extension at 72°C for 10 min. All the PCR amplicons were visualized using an ultraviolet light box after electrophoresis step to note specific bands at 776 bp (Figure1).

### 2.6. Sequencing step

PCR products of *mec*A gene of two multidrug-resistant MRSA isolates were purified using the QIAquick purification Kit (Qiagen, Germany) and direct cycle sequencing was performed on ABI 3500 Genetic Analyser (Applied Biosystems, USA) using Bigdye Terminator V3.1 cycle sequencing kit. The first software used in analysis of the sequencing result is DNA sequencing analysis software Version 5.1 for viewing and editing the sequence and the second one is SecScape V2.5 used for assembly of the all sequence reactions of the same sample and alignment.

### 2.7. Nucleotide sequence accession numbers

The GenBank nucleotide sequence accession numbers for partial sequences of *mec*A gene generated in this study were MN447540 and MN477948.

### 2.8. Phylogenetic analysis

**Figure 2.**
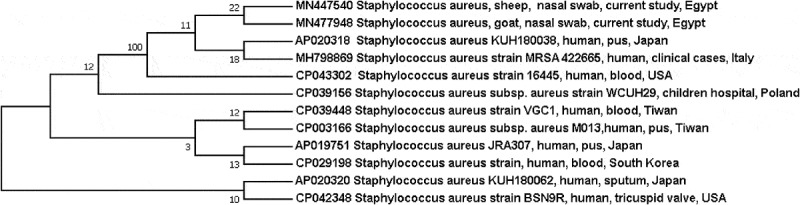
Phylogenetic consensus tree shows the evolutionary history of the obtained sequences. The analysis was carried out through neighbour-joining approach using Mega 7 software and based on the partial sequence of *mec*A gene.

The obtained sequences were compared with those available in the GenBank database through BLAST analysis. To investigate the public health threat of the obtained strains, sequences from the current study were aligned against the most similar ones of human cases retrieved from the Genbank using Clustal W multiple alignment by BioEdit software version (7.0.9). Phylogenetic tree was constructed through neighbour-joining approach using Mega7 software version 7.0.26 and bootstrap analysis was obtained with 500 replicates (Figure 2).

## 3. Results

MRSA isolates were recovered from 7 (3.6%) of 195 examined apparently healthy farm animals. The prevalence rates of MRSA among different animal species were 3.8%, 3.9%, 4.3% and 2.2% or sheep, goats, cattle and buffalo, respectively (Table1). All seven MRSA isolates exhibited a multidrug resistance. Penicillins and cefoxitin resistance were found in all isolates, while five of them were resistant to ceftaroline, azithromycin and quinupristin/dalfopristin. Moreover, erythromycin resistance was appeared in four ones, tetracyclines resistance in two strains, but only one isolate showed resistance to norfloxacin (Table 2).

**Table 1.** Distribution of MRSA among apparently healthy farm animals.

Animal species	No. of examined animals	No. of positive animals (%)
Sheep	52	2 (3.8)
Goats	51	2 (3.9)
Cattle	47	2 (4.3)
Buffalo	45	1 (2.2)
Total	195	7 (3.6)

**Table 2.** Antibiotic resistance pattern of obtained MRSA strains from different farm animals.

		Penicillins		Cephalosporins		Macrolides		Fluoroquinolones		Phenicols	Ansamycins	Oxazolidinones	Tetracyclines		Nitrofurantoins	Folate pathway antagonists	Aminoglycosides	Lincosamides	Streptogramins
Isolate No.	Species	(P)	(Ox)	(CX)	(CPT)	E	AZM	CIP	NX	C	RA	LZ	TE	DO	NIT	COT	GEN	CD	RP
1	**Cattle**	R	R	R	I	S	I	I	R	S	S	S	S	S	S	S	I	S	S
2	**Cattle**	R	R	R	R	I	R	S	S	S	S	S	R	R	S	S	I	S	R
3	**Sheep**	R	R	R	R	S	S	S	S	S	S	S	R	R	S	S	S	S	S
4	**Sheep**	R	R	R	R	R	R	S	S	S	S	S	S	S	S	S	S	S	R
5	**Buffalo**	R	R	R	R	R	R	S	S	S	S	S	S	S	S	S	S	S	R
6	**Goat**	R	R	R	R	R	R	S	S	S	S	S	S	S	S	S	S	S	R
7	**Goat**	R	R	R	S	R	R	S	S	S	S	S	S	S	S	S	S	S	R

Penicillin (**P**), Oxacillin (**Ox**), Cefoxitin (**CX**), Ceftaroline (**CPT**), Erythromycin (**E**), Azithromycin (**AZM**), Ciprofloxacin (**CIP**), Norfloxacin (**NX**), Chloramphenicol (**C**), Rifampin (**RA**), Linezolid (**LZ**), Tetracycline (**TE**), Doxycycline (**DO**), Nitrofurantoin (**NIT**), Trimethoprim/sulfamethoxazole (**COT**), Gentamicin (**GEN**), Clindamycin (**CD**), Quinupristin/dalfopristin (**RP**).

## 4. Discussion

Nowadays, emergence of antibiotic-resistant bacteria, especially MRSA is not only a global public health challenge but also an emerging veterinary pathogen throughout the world [21]. After the introduction of β-lactam antimicrobials, the prevalence of MRSA infections and colonization in food-producing animals has steadily increased over time [22,23]. In this study, the overall occurrence rate of MRSA among examined animals was 3.6% with the following rates among different animal species (sheep: 3.8%, goats: 3.9%, cattle: 4.3% and buffalo: 2.2%). The isolation rate in our study was lower than those recorded by Nemeghaire et al.19.8% [12] in the examined healthy bovines and Alzohairy 28.9%, 15.5%, 20% [24] in healthy sheep, cow and goats, respectively, but it was similar to that reported after examining cattle and calves in Switzerland 0.3% and 1% [25] and Danish small ruminants 1.5% [26]. On the contrary, all *S.aureus* isolates in the investigated apparently healthy animals from Tunisia [27] and China [28] were methicillin-susceptible *S. aureus* (MSSA).

MRSA is probably the best example of a prevalent and important multidrug-resistant bacterium that has successfully transitioned from an almost exclusively nosocomial setting to being widespread in the community [29]. All obtained MRSA isolates in this study were multidrug-resistant strains (exhibiting resistance to 3–5 antibiotic categories), a result which was in accordance with that reported in healthy bovines in Belgium [12] and healthy pigs in Southern Italy [13].

Unsurprisingly, results of MRSA isolate susceptibility testing showed 100% resistance to penicillin, oxacillin and cefoxitin; this result was in consistent with those obtained by Nemeghaire et al. and El-Deeb et al. [12,23]. It was noteworthy that five strains were resistant to ceftaroline. Penicillin binding protein 2a has a low affinity to beta-lactam antibiotics comprising penicillin, cephalosporin and carbapenems except the newely approved drug, ceftaroline [30]. Such drug, a fifth generation of cephalosporins, has approved by US Food and Drug Administration (FDA) in 2010 for the treatment of MRSA skin and soft tissue infections as well as it has been used to treat MRSA in refractory cases [31]. Therefore, detection of ceftaroline resistant MRSA in apparently healthy farm animals, alarms the public health community that MRSA becomes more resistant to advanced antibiotics, which may make it non curable in the future.

Although resistance to quinupristin-dalfopristin is rare among staphylococci in Unites states [32], five MRSA isolates from apparently healthy sheep, goats, cattle and buffalo in the current study were resistant to it. From a public health point of view, the widespread of resistance to such drug in different animal species is a great threat since quinupristin-dalfopristin can be considered as a treatment option for infections caused by MRSA, especially in patients intolerant or failing alternate therapy [33].

Furthermore, 5, 4 and 2 MRSA isolates in the current study showed resistance to azithromycin, erythromycin and tetracyclines, respectively. These antibiotics are of human and veterinary concern because they are widely used in human and veterinary medicine. On the other hand, all isolates were susceptible to linezolid, chloramphenicol, rifampin, trimethoprim/sulfamethoxazole, nitrofurantoin and clindamycin.

The findings of the current study underscore the circulation of multidrug-resistant MRSA strains among apparently healthy farm animals and accordingly, farm animals may be considered as a reservoir for such multidrug-resistant strains which may easily pass to humans through direct contact to initiate a horizontal transmission of these strains between humans a matter which has a great public health burden [34]. The phylogenetic analysis of *mec*A gene sequences of two isolates revealed that both sequences were grouped in the same clade with those obtained from human cases in USA, Japan and Italy. Moreover, they were closely related to hospital-acquired MRSA strain from Children’s University Hospital in Warsaw, Poland (accession no. CP039156) to point out the public health significance of such isolates.

## 5. Conclusion

Our findings reported the occurrence of multidrug-resistant MRSA among apparently healthy farm animals to highlight the potential role of such animals in the epidemiology of multidrug-resistant MRSA strains which may act as a reservoir for human infections.
